# The Crucial Role of Xanthine Oxidase in CKD Progression Associated with Hypercholesterolemia

**DOI:** 10.3390/ijms21207444

**Published:** 2020-10-09

**Authors:** You-Jin Kim, Se-Hyun Oh, Ji-Sun Ahn, Ju-Min Yook, Chan-Duck Kim, Sun-Hee Park, Jang-Hee Cho, Yong-Lim Kim

**Affiliations:** 1 Division of Nephrology, Kyungpook National University Hospital, Daegu 41944, Korea; pinkqic1004@naver.com (Y.-J.K.); ttily@nate.com (S.-H.O.); ggumsuni@hanmail.net (J.-S.A.); jumin18@hanmail.net (J.-M.Y.); drcdkim@mail.knu.ac.kr (C.-D.K.); sh-park@knu.ac.kr (S.-H.P.); 2 Cell and Matrix Research Institute, Kyungpook National University, Daegu 41944, Korea; 3 Department of Internal Medicine, School of Medicine, Kyungpook National University, Daegu 41944, Korea

**Keywords:** chronic kidney disease, hypercholesterolemia, xanthine oxidase, inflammation, fibrosis, NF-κB pathway

## Abstract

In the present study, we investigated the effects of xanthine oxidase (XO) inhibition on cholesterol-induced renal dysfunction in chronic kidney disease (CKD) mice, and in low-density lipoprotein (LDL)-treated human kidney proximal tubule epithelial (HK-2) cells. ApoE knockout (KO) mice underwent uninephrectomy to induce CKD, and were fed a normal diet or high-cholesterol (HC) diet along with the XO inhibitor topiroxostat (1 mg/kg/day). HK-2 cells were treated with LDL (200 µg/mL) and topiroxostat (5 µM) or small interfering RNA against xanthine dehydrogenase (siXDH; 20 nM). In uninephrectomized ApoE KO mice, the HC diet increased cholesterol accumulation, oxidative stress, XO activity, and kidney damage, while topiroxostat attenuated the hypercholesterolemia-associated renal dysfunction. The HC diet induced cholesterol accumulation by regulating the expressions of genes involved in cholesterol efflux (*Nr1h3* and *Abca1*) and synthesis (*Srebf2* and *Hmgcr*), which was reversed by topiroxostat. Topiroxostat suppressed the expressions of genes related to hypercholesterolemia-associated inflammation and fibrosis in the unilateral kidney. LDL stimulation evoked changes in the cholesterol metabolism, nicotinamide adenine dinucleotide phosphate (NADPH) oxidase, and NF-κB pathways in HK-2 cells, which were mitigated by XO inhibition with topiroxostat or siXDH. These findings suggest that XO inhibition exerts renoprotective effects against hypercholesterolemia-associated kidney injury. XO could be a novel therapeutic target for hypercholesterolemia-associated kidney injury in uninephrectomized patients.

## 1. Introduction

Compared to people with normal cholesterol levels, those with high cholesterol levels are 1.5 times more likely to develop kidney dysfunction [1,2]. Individuals with metabolic syndrome who serve as kidney donors reportedly show a higher incidence of post-donation kidney disease due to dyslipidemia and obesity compared to normal donors [3,4]. These findings suggest that abnormal cholesterol metabolism independently increases the risk of chronic kidney disease.

Dyslipidemia may directly affect the kidney by causing deleterious renal lipid disturbances, and indirectly affect the kidney through systemic inflammation, oxidative stress and vascular injury [5,6]. We previously reported that high concentrations of uric acid synthesis metabolites lead to the generation of reactive oxygen species (ROS), which promotes hypercholesterolemia with cholesterol accumulation in hepatocytes and incites atherosclerosis in apolipoprotein E (ApoE)-deficient mice [7]. However, the effects of cholesterol on the kidneys have not been confirmed.

In a unilateral kidney, a high-fat diet alters the expressions of genes involved in the cytoskeletal remodeling, fibrosis, and oxidative stress pathways. This suggests that the high-fat diet has synergistic effects that promote gene expression changes related to kidney damage [8]. Additionally, in animal experiments, unilateral kidney dyslipidemia is associated with the exacerbation of kidney damage, characterized by decreased kidney function, vasodilation, fibrosis, oxidative stress, and ER stress, despite normal body weight [9]. These findings suggest that kidney lipid accumulation and lipid toxicity play important roles in the pathogenesis of kidney damage, and are associated with a higher risk of kidney damage in unilateral kidneys.

It has been postulated that oxidative stress—derived from nicotinamide adenine dinucleotide phosphate (NADPH) oxidase, mitochondrial oxidase, and xanthine oxidase (XO)—is involved in renal lipid disturbances [10]. Notably, high cholesterol levels activate XO and lead to excessive ROS formation, resulting in tissue damage, such as inflammation and atherosclerosis [11,12]. Consistently, XO inhibitors exhibit pronounced protective effects against vascular injuries, inflammatory diseases, and tissue damage [13,14]. XO inhibitors can also inhibit the expression of pro-inflammatory proteins. Therefore, XO inhibitors have potential for use as therapeutic drugs that reduce oxidative stress [15]. However, the XO inhibitor allopurinol does not inhibit renal dysfunction in patients with chronic kidney disease (CKD) and high risk of progression [16].Thus, the effect of XO inhibitors in terms of kidney protection remains controversial.

In the present study, we investigated the correlation between cell injury and non-oxidized LDL-induced ROS in vitro, as well as the effects of a high-cholesterol (HC) diet and hypercholesterolemia on lipid accumulation and renal tubule damage in vivo using a unilateral kidney model. We further aimed to identify potential signaling pathways that could be affected by lipid accumulation-induced xanthine oxidase and ROS under a CKD associated with hypercholesterolemia.

## 2. Results

### 2.1. Hypercholesterolemia Aggravates Renal Function Through Increased Kidney Lipid Accumulation, Xanthine Oxidase Activity, and Oxidative Stress

**Figure 1:**
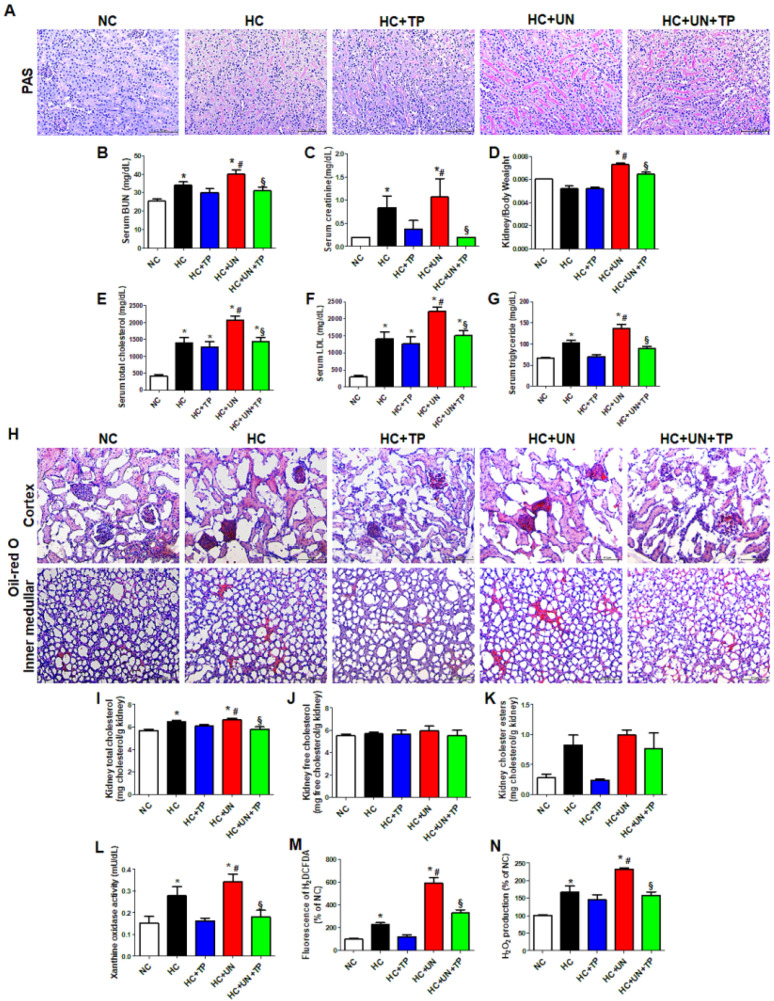
The effects of xanthine oxidase inhibition on high-cholesterol diet-induced cholesterol accumulation, oxidative stress, and damage in the kidney. (**A**) Representative images of periodic acid Schiff (PAS) staining of kidney tissue. Scale bars, 100 μm. (**B**–**D**) Levels of blood kidney injury factors (BUN and creatinine) and kidney/body weight in mice from different groups. (**E**–**G**) Serum cholesterol levels in mice from different groups. (**H**) Representative images of Oil red O staining of kidney tissue (upper: cortex, lower: inner-medullar). Scale bars, 100 μm. (**I**–**K**) Total cholesterol, free cholesterol, and cholesterol ester levels in the kidney. (**L**–**N**) Xanthine oxidase activity, DCFDA, and H_2_O_2_ production in kidney tissue. Differences among the groups were analyzed by a one-way non-parametric ANOVA, followed by Tukey’s multiple comparison test. Data represent mean and SEM. * *p* < 0.05 vs. NC, # *p* < 0.05 vs. HC, § *p* < 0.05 vs. HC+UN. TP, topiroxostat; UN, uninephrectomy; NC, normal control; HC, high-cholesterol.

PAS staining confirmed that the high-cholesterol (HC) diet led to renal tubular damage in the unilateral kidney (Figure 1A). The HC diet yielded increased serum blood urea nitrogen (BUN) and creatinine, which were highest with HC+uninephrectomy (UN) group (Figure 1B–C). The XO inhibitor topiroxostat (TP) attenuated this kidney dysfunction (Figure 1A–C). Kidney weight was increased in uninephrectomized mice, whereas reduced by TP treatment (Figure 1D). In both sham-operated and UN mice, HC diet yielded increased serum total cholesterol (TC), low-density lipoprotein (LDL), and triglycerides (TG), and TP treatment restored these levels (Figure 1E–G).

Compared to normal control (NC), HC and HC+UN yielded increased lipid droplets in the kidney, showing higher levels with HC+UN versus HC (Figure 1H). Kidney TC was higher with HC and HC+UN versus NC, and was reduced by TP in the HC+UN+TP group (Figure 1I). Free cholesterol did not differ among groups. HC and HC+UN showed trends of increased kidney cholesterol esters (Figure 1J,K). TP decreased kidney cholesterol accumulation, consistent with decreased serum TC, LDL, and TG. The HC diet significantly elevated XO activity, dichlorodihydrofluorescein diacetate (DCFDA), and H_2_O_2_ excretion in kidney tissue, which were higher with HC+UN versus HC. TP decreased the increased XO activity and intracellular oxidative stress markers (Figure 1L–N).

### 2.2. Effects of Hypercholesterolemia on Genes Related to Cellular Cholesterol Transport and Synthesis in the Unilateral Kidney

**Figure 2:**
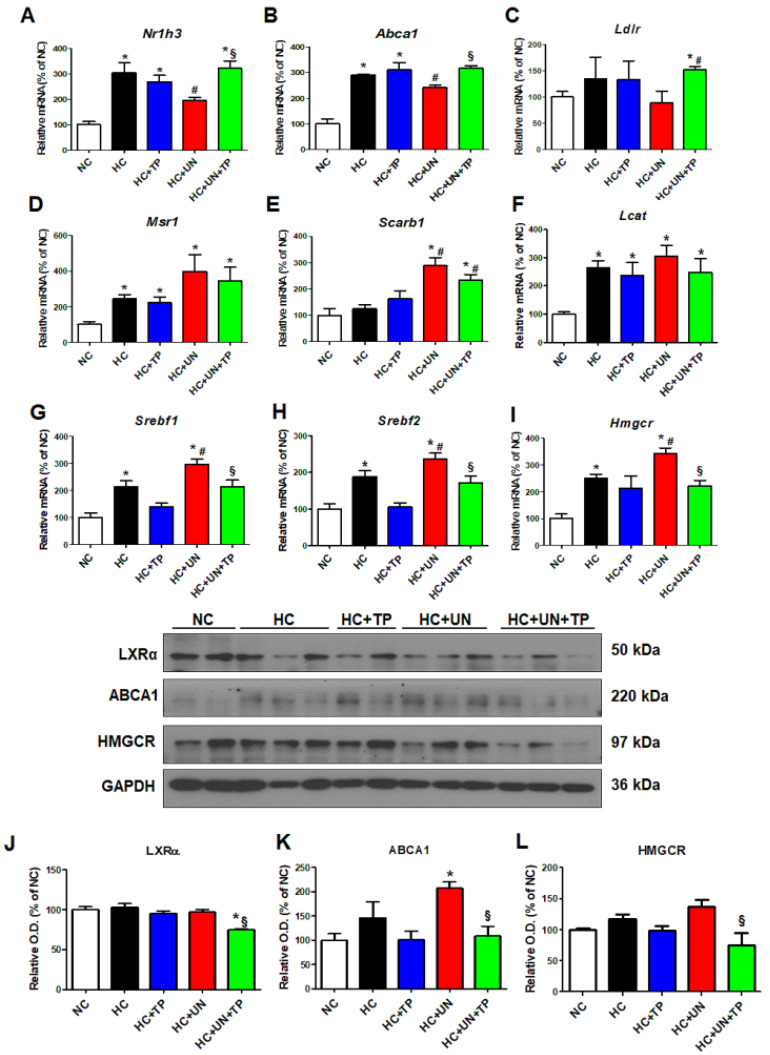
TP decreased cholesterol accumulation in the kidney through regulation of genes involved in cholesterol transport and synthesis. Real-time RT-PCR was performed to determine the expression levels of genes involved in cholesterol efflux (**A**,**B**), LDL receptor (**C**), modified LDL influx transport (**D**,**E**), cholesterol esterase (**F**), triglyceride synthesis (**G**), and cholesterol synthesis (**H**,**I**). (**J**–**L**) Representative western blot for LXRα, ABCA1, HMGCR, and GAPDH. Relative protein expression was determined using densitometry. Differences among the groups were analyzed by one-way non-parametric ANOVA, followed by Tukey’s multiple comparison test. Data represent mean and SEM. * *p* < 0.05 vs. NC, # *p* < 0.05 vs. HC, § *p* < 0.05 vs. HC+UN. LDL, low-density lipoprotein; TP, topiroxostat; UN, uninephrectomy; NC, normal control; HC, high-cholesterol.

Compared to NC, the HC diet groups exhibited increased mRNA expression of genes related to cholesterol efflux (*Nr1h3* and *Abca1*), LDL and modified LDL uptake (*Ldlr*, *Msr1*, and *Scarb1*), TG synthesis (*Srebf1*), and cholesterol synthesis (*Srebf2* and *Hmgcr*) (Figure 2A–I). HC+UN yielded decreased *Nr1h3* and *Abca1* (Figure 2A,B), increased *Msr1* and *Scarb1*, and decreased *Ldlr* expression (Figure 2C–E). *Lcat* expression was increased with the HC diet versus normal diet but did not differ between HC and HC+UN (Figure 2F). HC+UN yielded increased *Srebf1*, *Srebf2*, and *Hmgcr* expression (Figure 2G–I). TP treatment (HC+UN+TP) increased *Nr1h3* and *Abca1* expression, and decreased *Srebf1*, *Srebf2*, and *Hmgcr* expression (Figure 2A,B,G–I). The HC diet led to increased ABCA1 and HMGCR protein levels, which were higher with HC+UN versus HC. LXRα protein levels were not altered by HC diet (Figure 2J–L). Overall, TP treatment yielded increased cholesterol efflux gene expression and reduced cholesterol synthesis gene expression (Figure 2).

### 2.3. Effects of LDL on Cholesterol Metabolism and Oxidative Stress in HK-2 Cells

**Figure 3:**
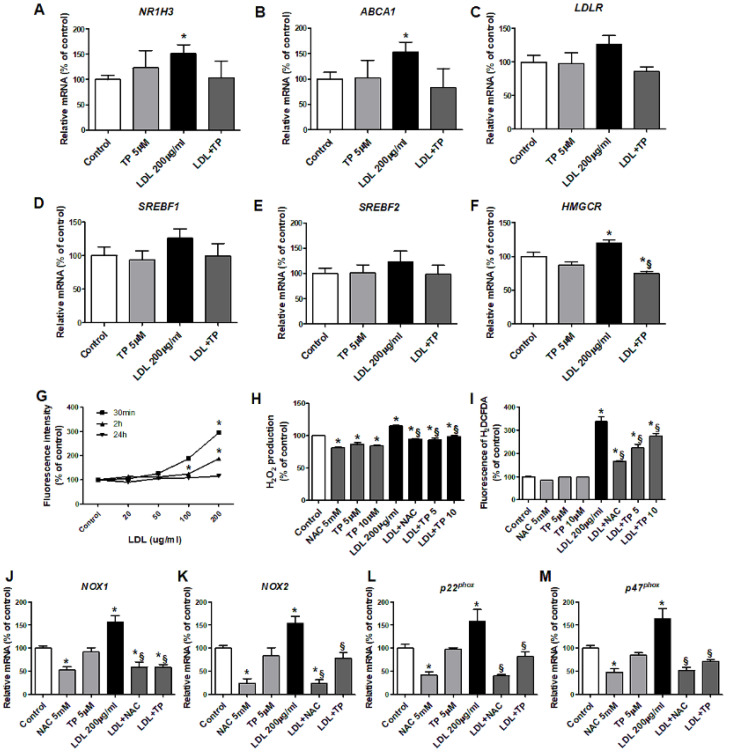
Effects of xanthine oxidase inhibitor on the expression of genes related to cholesterol transport and synthesis and ROS in LDL-stimulated HK-2 cells. Real-time RT-PCR was performed to determine the expression levels of genes related to cholesterol efflux (**A**,**B**), the LDL receptor (**C**), triglyceride synthesis (**D**), and cholesterol synthesis (**E**,**F**). (**G**) After pretreatment with TP (5 or 10 μM) for 1 h, and stimulation with LDL (200 μg/mL) for 30 min, cellular ROS generation was measured based on the fluorescence intensity of DCF. (**H**,**I**) Measurement of H_2_O_2_ and DCFDA production. Quantitative PCR was performed to analyze the relative mRNA expression of *NOX1* (**J**), *NOX2* (**K**), and the NADPH oxidase subunits *p22^phox^* (**L**), *p47^phox^* (**M**). Differences among the groups were analyzed by one-way non-parametric ANOVA, followed by Tukey’s multiple comparison test. NAC was used as a positive control. Data represent mean and SEM. * *p* < 0.05 vs. Control, § *p* < 0.05 vs. LDL. TP, topiroxostat; LDL, low-density lipoprotein; DCF, dichlorofluorescein.

LDL increased *NR1H3*, *ABCA1*, and *HMGCR* expression in human kidney proximal tubule epithelial (HK-2) cells, without affecting *LDLR*, *SREBF1*, or *SREBF2*. TP decreased only *HMGCR* expression (Figure 3A–F). LDL increased intracellular ROS over the basal level, which peaked at 30 min (Figure 3G), with elevated H_2_O_2_ level and ROS-dependent DCF fluorescence intensity. TP reduced cellular ROS, similar to the positive control NAC (Figure 3H–I). 

**Figure 4:**
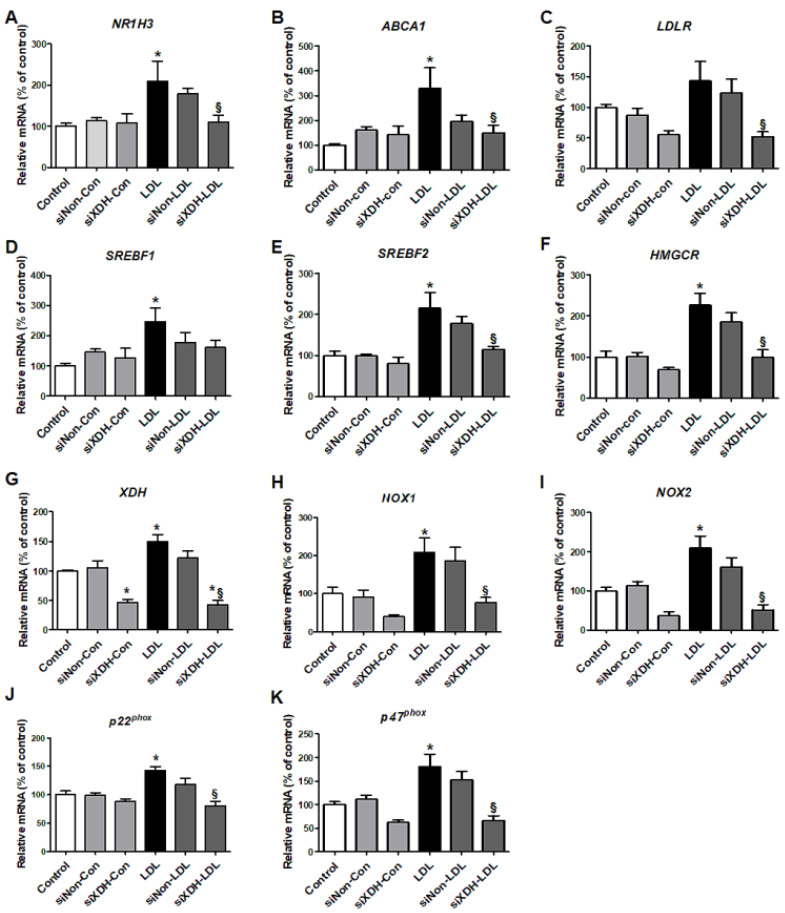
The effects of small interfering RNA against XDH (siXDH) on cholesterol metabolism and oxidative stress in LDL-stimulated HK-2 cells. After transfection with siXDH (20 μM) for 24 h, and stimulation with LDL (200 μg/mL) for 30 min, real-time RT-PCR was performed to determine expression levels of cholesterol efflux genes (**A**,**B**), LDL receptor (**C**), triglyceride synthesis (**D**), cholesterol synthesis (**E**,**F**), *XDH* (**G**), *NOX1* (**H**), *NOX2* (**I**), and the NADPH oxidase subunits *p22^phox^* (**J**) and *p47^phox^* (**K**). Differences among the groups were analyzed by one-way non-parametric ANOVA, followed by Tukey’s multiple comparison test. Data represent mean and SEM. * *p* < 0.05 vs. control, § *p* < 0.05 vs. LDL. LDL, low-density lipoprotein.

Using siXDH, we evaluated how XO knock-down influenced cholesterol-related metabolism. LDL increased *XDH* expression in HK-2 cells (Figure 4G), and induced expression of the cholesterol transport genes *NR1H3* and *ABCA1*, but not *LDLR*. The siXDH reduced transport gene expression in LDL-treated HK-2 cells, to a greater extent than XO inhibitor (Figure 4A–C). XO deficiency regulated cholesterol synthesis genes upon LDL stimulation. LDL stimulation increased expressions of *SREBF1*, *SREBF2*, and *HMGCR*, while siXDH suppressed the increased expressions of *SREBF2* and *HMGCR* (Figure 4D–F). 

We examined mRNA expressions of subunits of the ROS generation enzyme NADPH oxidase. LDL-treated HK-2 cells exhibited increased mRNA expressions of *NOX1*, *NOX2*, *p22^phox^*, and *p47^phox^*. TP inhibited these increased expressions, similar to NAC (Figure 3J–M). siXDH transfection also reduced expression of *NOX1*, *NOX2*, *p22^phox^*, and *p47^phox^* (Figure 4H–K).

### 2.4. XO Inhibition Reduces Hypercholesterolemia-Associated Kidney Inflammation and Fibrosis in CKD Mice

**Figure 5:**
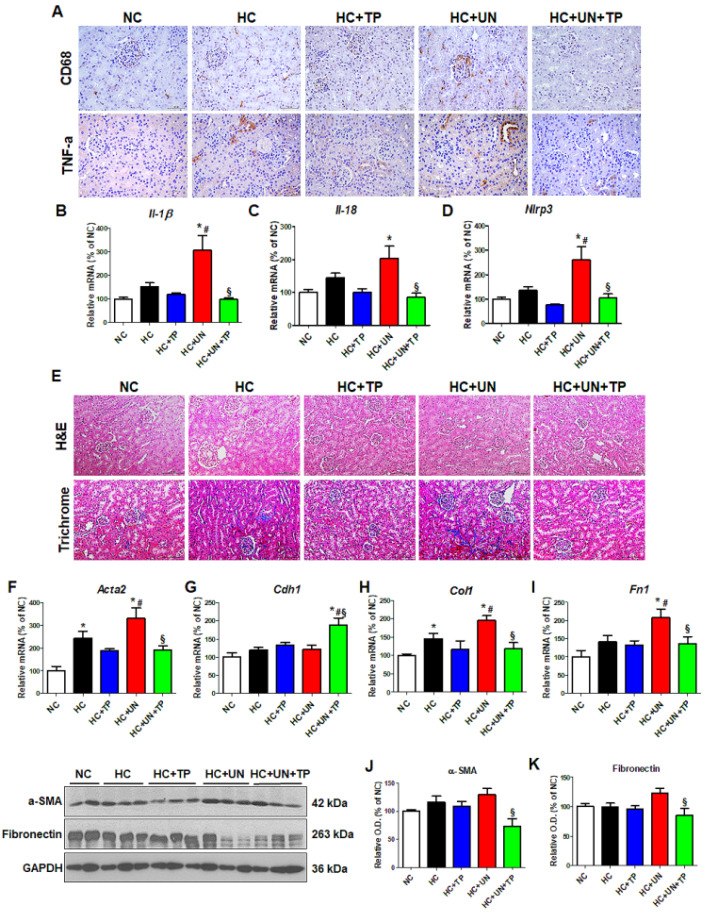
Abnormal lipid metabolism and oxidative stress induced by high-cholesterol diet affect inflammation and fibrosis progression in kidney tissue. (**A**) Representative images of TNF-α and CD68 staining of kidney tissue. Scale bars, 100 μm. (**B**–**D**) Real-time RT-PCR was performed to determine mRNA levels of *Il-1β*, *Il-18*, and *Nlrp3*. (**E**) Representative images of hematoxylin and eosin staining and trichrome staining of kidney tissue. Scale bars, 100 μm. (**F**–**I**) Real-time RT-PCR was performed to determine mRNA levels of *Acta2*, *Cdh1*, *ColI*, and *Fn1*. (**J**,**K**) Representative western blot for α-SMA, fibronectin, and GAPDH. Relative protein expression was determined using densitometry. Differences among the groups were analyzed by one-way non-parametric ANOVA, followed by Tukey’s multiple comparison test. Data represent mean and SEM. * *p* < 0.05 vs. NC, # *p* < 0.05 vs. HC, § *p* < 0.05 vs. HC+UN. LDL, low-density lipoprotein; TP, topiroxostat; UN, uninephrectomy; NC, normal control; HC, high-cholesterol.

We evaluated the inflammatory responses to high cholesterol and oxidative stress in the kidney of a CKD model. The HC diet increased inflammation cytokines (e.g., TNF-α) and the macrophage monocyte marker CD68, which were higher with HC+UN versus HC (Figure 5A). Expressions of the inflammation-related genes *Il-1β*, *Il-18*, and *Nlrp3* were also increased with HC+UN versus HC. TP reduced inflammation-related gene expression in uninephrectomized mice (Figure 5B–D). Compared to NC, HC groups showed progression in the fibrosis of glomeruli and interstitium, which was greatest with HC+UN, and was reduced by TP (Figure 5E). Compared to HC, HC+UN yielded increased *Acta2*, *Col1*, and *Fn1* expressions (Figure 5F–I). Fibronectin and α-SMA protein expressions were not significantly increased in the HC+UN group (Figure 5J,K). 

**Figure 6:**
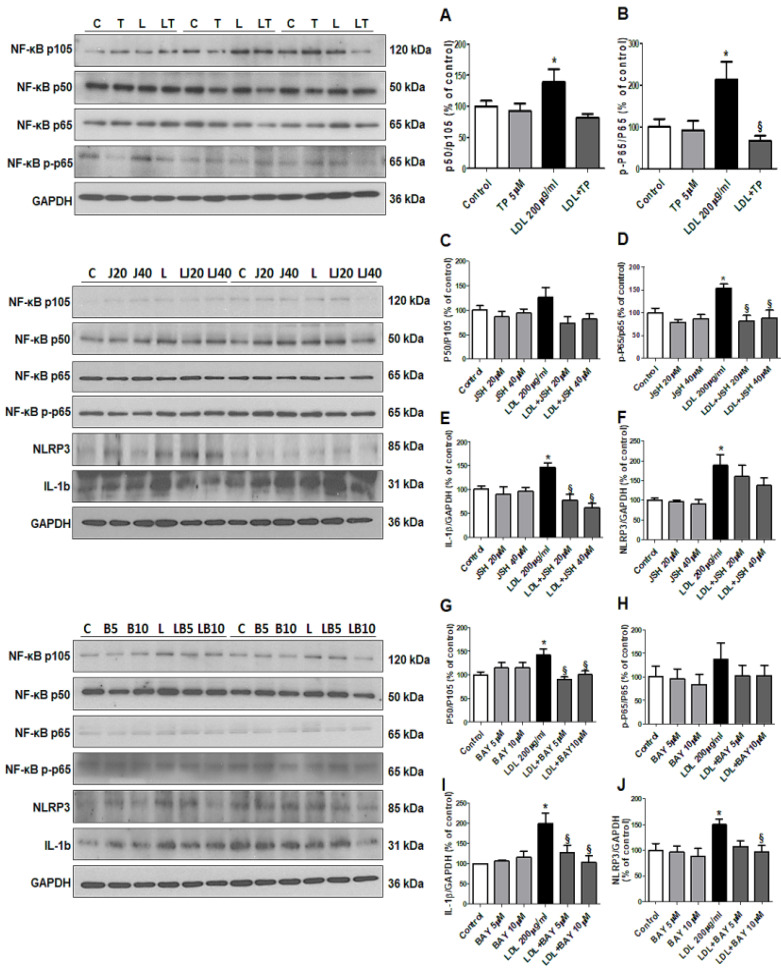
Effects of xanthine oxidase inhibitor on progression of inflammation through the NF-κB pathway in LDL-treated HK-2 cells. (**A**,**B**) Representative western blot for NF-κB p50/p105 ratio, NF-κB p-p65/p65 ratio, and GAPDH in cells that were pretreated with TP (5 μM) for 1 h, and then stimulated with LDL (200 μg/mL) for 30 min. (**C**–**F**) Representative western blot for NF-κB p50/p105 ratio, NF-κB p-p65/p65 ratio, IL-1b, NLRP3, and GAPDH in cells that were pretreated with NF-κB inhibitor (JSH23) for 1 h, and then stimulated with LDL (200 μg/mL) for 30 min. (**G**–**J**) Representative western blot for NF-κB p50/p105 ratio, NF-κB p-p65/p65 ratio, IL-1b, NLRP3, and GAPDH in cells that were pretreated with NF-κB inhibitor (BAY11-7028) for 2 h, and then stimulated with LDL (200 μg/mL) for 30 min. Relative protein expression was determined using densitometry. Differences among the groups were analyzed by a one-way non-parametric ANOVA, followed by Tukey’s multiple comparison test. Data represent mean and SEM. * *p* < 0.05 vs. Control, § *p* < 0.05 vs. LDL. LDL, low-density lipoprotein; TP, topiroxostat.

Finally, we evaluated the effects of LDL on the NF-κB pathway [17], downstream of TNF-α. LDL-stimulated HK-2 cells showed increased NF-κB p50 and NF-κB p65 levels, which were decreased by TP (Figure 6A–B). We also investigated the NF-κB inhibitors JSH23 and BAY11-7082. In LDL-treated HK-2 cells, JSH23 treatment significantly decreased NF-κB p65, and IL-1β, but did not affect NLRP3 and NF-κB p50 (Figure 6C–F), while BAY11-7082 treatment reduced NF-κB p50, IL-1β, and NLRP3 levels, but did not affect NF-κB p65 (Figure 6G–J).

## 3. Discussion

The present results demonstrated that XO inhibition significantly reduced hypercholesterolemia-associated kidney inflammation and fibrosis in uninephrectomized ApoE KO mice. In vitro experiments revealed that XO inhibition reduced LDL-induced oxidative stress and cholesterol synthesis. The HC diet induced cholesterol accumulation by regulating the expression of genes related to cholesterol efflux and synthesis. Additionally, hypercholesterolemia significantly increased XO activity and NADPH-dependent ROS generation, thus inducing kidney inflammation and fibrosis. LDL stimulation evoked changes in the cholesterol metabolism, NADPH oxidase, and NF-κB pathways in HK-2 cells. XO inhibition modulated the hypercholesterolemia-induced changes in the expression of genes related to cholesterol transport and synthesis. XO inhibition by topiroxostat attenuated inflammation and fibrosis in the unilateral kidney through inhibition of the NF-κB pathway.

Patients with CKD exhibit various lipid abnormalities [18], and abnormal lipid metabolism is associated with kidney disease progression [19,20]. However, the mechanism of lipid-induced kidney damage is not fully understood. In the present study, we used ApoE KO mice with unilateral nephrectomy as a model to evaluate the effects of hypercholesterolemia on CKD progression. Hypercholesterolemia aggravated renal function in the uninephrectomized ApoE KO mice by increasing kidney lipid accumulation. Moreover, these lipid abnormalities were associated with increases of factors mediating kidney injury, such as XO and oxidative stress.

Our results demonstrated that cholesterol accumulation in uninephrectomized mice was induced by upregulation of genes related to cholesterol synthesis, such as *Srebf2* and *Hmgcr,* and downregulation of efflux-related genes, such as *Abca1* and *Nr1h3*, upstream of ABCA1. Cholesterol homeostasis is regulated by multiple pathways, including intracellular cholesterol uptake, synthesis, and efflux actions [21]. Inflammatory stress promotes reduction of cholesterol efflux through the ABCA1 pathway, leading to lipid accumulation in the kidney [22]. ApoE promotes cholesterol efflux through the ABCA1 and ABCG1 cell surface transporters, which facilitate the efflux of phospholipids and cholesterol onto lipid-poor apolipoproteins [23]. The ABCA1 transporter facilitates the efflux of cellular phospholipids and cholesterol to acceptors, such as ApoA-I and ApoE [24]. Selective inactivation of macrophage ABCA1 yields substantially increased cholesterol accumulation in mice [25,26]. Our present results showed that the HC diet caused upregulated expression of both cholesterol synthesis and efflux genes. Furthermore, uninephrectomy plus hypercholesterolemia attenuated the increase of cholesterol efflux genes, while upregulating the increase of cholesterol synthesis genes. It is not yet known why uninephrectomy had different effects on the expression of genes related to cholesterol metabolism. However, our findings suggest that hypercholesterolemia-associated genes contributed to cholesterol accumulation in the kidney, and may potentially play a role in the dysfunction of the unilateral kidney.

The ABCA1 protein level differed from mRNA expression in the unilateral kidney with high cholesterol. The ABCA1 membrane protein rapidly responds to increased cholesterol, and the ABCA1 protein level is independent of its transcription [27]. Cholesterol accumulation inhibits ABCA1 degradation and ubiquitination by decreasing the proteasomal degradation [28]. In our study, the decrease of *Abca1* mRNA may have been associated with the reduced intracellular cholesterol efflux, and thus with the increased cholesterol accumulation in the kidney. 

Abnormal lipid metabolism induces monocyte foam cells, and this change is more prominent in patients with kidney dysfunction [29]. Alterations in lipoprotein metabolism promote the production of ROS, such as hydrogen peroxide; however, the effect on intracellular cholesterol has not been clearly demonstrated. Since increased serum LDL level is a representative marker of dyslipidemia, we stimulated renal tubular cells with LDL to examine the molecular changes in cholesterol metabolism and inflammation. LDL stimulation increased the mRNA expression of *NR1H3*, *ABCA1*, *LDLR*, *SREBF2*, and *HMGCR* in HK-2 cells. These changes are consistent with our observations in the animal model, except for the expression of *SREBF1*, which is involved in TG synthesis. These findings suggest that the renal tubular cell damage is also mediated by the lipid accumulation associated with changes in the expression of genes related to cholesterol transport and synthesis. 

XO is a key enzyme of the purine pathway, producing uric acid through various oxidized purines [30]. Recent studies demonstrate that XO plays a role as an ROS-producing enzyme, aggravating inflammation, atherosclerosis, and chronic disease [31]. XO activity is increased in obesity, and XO inhibitors regulate the inflammatory process by inhibiting production of ROS and uric acid [32,33]. We previously demonstrated that XO inhibition could decrease cancer cell migration and ROS generation [10]. In our present study, we found that XO activity and gene expression were increased in kidneys with hypercholesterolemia, and in LDL-treated HK-2 cells. This is the first report of cholesterol directly leading to increased XO enzyme activity in the kidney. We further found that XO inhibition improved hypercholesterolemia-associated kidney damage by modulating the expression of genes related to cholesterol metabolism and the progression of inflammation and fibrosis in a unilateral kidney. Overall, our findings suggest that XO is a promising treatment target for hypercholesterolemia-associated kidney injury in uninephrectomized patients. 

Expression of NOX1, NOX2, and NOX4 in the kidney mediates oxidative stress and promotes vascular inflammation, dysfunction, and fibrosis in CKD [34]. NADPH oxidases of the kidney exhibit a distinct cellular localization and are activated by various stimuli, including LDL [35]. Our results demonstrated that hypercholesterolemia and LDL increased oxidative stress in kidney tissue and tubule cells, and that XO activity was associated with oxidative stress markers and increased expression of NADPH-related genes. XO inhibition reduced the expression of *NOX1* and *NOX2*, but not *NOX4* (data not shown), showing antioxidant effects similar to with NAC. Transfection with siXDH had the same effect as XO inhibitor in HK-2 cells. These results revealed that XO inhibition controlled NOX activity, thus suppressing oxidative stress and reducing renal damage. 

The inflammatory process exacerbates lipid accumulation in the kidney by translocating plasma lipids to the kidney [36]. A high-fat diet activates the inflammatory response by increasing TNF-α expression in the kidney, thus causing kidney damage in obese humans and animal models [37,38]. XO activity is elevated in inflammation, and XO inhibition suppresses inflammation and oxidative stress in macrophages [14]. Treatment with XO inhibitors reportedly attenuates inflammation and fibrosis in animal models of atherosclerosis and nonalcoholic steatohepatitis [39]. Here we found that a HC diet led to increased TNF-α and CD68 in the unilateral kidney, which was downregulated after XO inhibition. These results showed that the mechanism of hypercholesterolemia-associated kidney damage is closely related to inflammation, and that the damage could be suppressed by XO inhibition.

Oxidative stress stimulates inflammatory cells to activate the NF-κB pathway through an intracellular signaling system, thus mediating inflammation [40]. In cells, oxidative stress induces NLRP3 activity, leading to inflammation and apoptosis, along with Caspase-1 activation. Obesity and metabolic syndrome induce NLRP3 inflammatory activation, which weakens phospholipid degradation, leading to kidney damage [41]. In this study, hypercholesterolemia increased NLRP3 and TNF-α, which is one of the most potent inducers of NF-κB. In vitro experiments demonstrated that the NF-κB pathway was an inflammatory mechanism of dyslipidemia-associated kidney damage. LDL stimulation increased the activity of the NF-κB pathway and NLRP3, whereas XO inhibition affected the NF-κB pathway by decreasing phosphorylated NF-κB p65 and NF-κB p50 in HK-2 cells. We further demonstrated that the NF-κB inhibitors JSH23 and BAY11-7028 had effects on NLRP3 similar to those of XO inhibition. This supports the theory that NF-κB is a main pathway of hypercholesterolemia-associated kidney damage.

In conclusion, our present data suggested that XO inhibition has renoprotective effects against hypercholesterolemia-associated kidney injury. This protection from XO inhibition was mediated by regulation of cholesterol metabolism, decreasing NADPH-dependent ROS generation, and reduction of inflammation through the NF-κB pathway. XO could be a novel therapeutic target for hypercholesterolemia-associated kidney disease in uninephrectomized patients.

## 4. Materials and Methods

### 4.1. Animals, Diets, and Specimen Collections

Male ApoE KO mice were purchased from Jackson Laboratory (Bar Harbor, ME, USA) at the age of 8 weeks. All animal experiments were approved and performed according to the regulations of the Kyungpook National University Animal Care and Use Committee (KNU-2018-0042, date; 08/03/2018). For 12 weeks, animals were fed normal control diet containing 20.3% protein, 5% fat, and 66% carbohydrate or a high-fat high-cholesterol diet (HC, diet D12336; Research Diets, New Brunswick, NJ, USA) containing 16.0% fat, 1.25% cholesterol, and 0.5% sodium cholic acid.

We randomized the eight-week-old ApoE KO mice into groups that did or did not receive topiroxostat (TP; 1 mg per kg body weight) treatment, and that did or did not undergo uninephrectomy (UN) surgery. There were a total of five groups: NC (*n* = 5), fed a normal control diet; HC (*n* = 6), fed a high-cholesterol diet; HC+TP (*n* = 6), fed a high-cholesterol diet with TP; HC+UN (*n* = 8), fed a high-cholesterol diet and received uninephrectomy surgery; and HC+UN+TP (*n* = 8), fed a high-cholesterol diet with TP and received uninephrectomy surgery. All mice that did not undergo uninephrectomy were sham operated. TP was administered by oral gavage for 4 weeks before the end of the experiment.

Uninephrectomy was performed as described previously [42]. For uninephrectomy, after mice were anesthetized with 3–5% isoflurane, the left kidney was surgically removed via a left incision on the back. The adrenal gland was carefully freed from the upper pole of the renal capsule before removed the left kidney. The incision was closed with sutures. In sham surgery, the kidney was manipulated without ablation. At 12 weeks after surgery, all mice were anesthetized and then sacrifice, and kidneys were harvested for the analyses. Half of the kidneys were stored at −80 °C for molecular analysis and the other half was fixed with 4% paraformaldehyde for histological analysis. 

### 4.2. Serum Chemistry

At the end of the experimental period, blood samples from each mouse were collected into tubes by cardiac puncture. The blood was sampled into ethylenediaminetetraacetic acid-free bottles for serum separation. The blood urea nitrogen (BUN), creatinine (Cr), uric acid, total cholesterol (TC), low-density lipoprotein (LDL) and triglycerides level in the serum were measured by GCLabs (Yongin, Korea) using the Cobas 8000 modular analyzer system (Roche, Basel, Switzerland).

### 4.3. Histopathology 

To detect kidney injury, the fixed right kidney was dehydrated in ethanol and embedded in paraffin. Kidney tissue blocks were cut into 2-μm-thick sections and subjected to hematoxylin and eosin (H&E) staining, periodic acid Schiff (PAS) staining, and Masson’s trichrome staining. For immunohistochemical analysis of kidney tissues, we used the following antibodies: mouse monoclonal against CD68 (ED1; 1:100; Abcam, Cambridge, MA, USA) and rabbit polyclonal against tumor necrosis factor-α (TNF-α; 1:200; Abcam). Next, secondary antibody was performed using HRP-conjugated polyclonal goat anti-rabbit IgG P0447 or goat anti-mouse IgG p0448 (Dako, Glostrup, Denmark) for 1 h. The sections were visualized using 3,3-diaminobenzidine (DAB; DAKO ChemMate Detection Kit) and counterstained with Mayer’s hematoxylin. 

### 4.4. Oil Red O Staining 

Fixed frozen kidney tissue was cut into 6-μm-thick sections, and subjected to Oil Red O (Sigma-Aldrich, Saint Louis, MO, USA) staining following the manufacturer’s protocol. After the sections were rinsed in distilled water and 60% isopropanol for 1 min, the sections were stained with Oil red O for 15 min. and then rinsed in 60% isopropanol and distilled water each for 1 min. Counterstaining was stained with hematoxylin for 1 min. 

### 4.5. Cell Treatments 

Human renal proximal tubule epithelial cells (HK-2 cells) were purchased from the Korean Cell Line Bank (KCLB, Seoul, South Korea). The cells were maintained in RPMI-1640 supplemented with 10% fetal bovine serum, and 100 units/mL penicillin and 100 μg/mL streptomycin antibiotic mixture at 37 °C in 5% CO_2_ and 95% air. The Cells were pretreated with topiroxostat (TP), xanthine oxidase inhibitor (5 or 10 µM), and 5 mM N-acetylcysteine (NAC) (Sigma-Aldrich) for 1 h, and stimulated with LDL (200 µg/mL) for 30 min to induce lipotoxicity. 

### 4.6. Hydrogen Peroxide Determination

H_2_O_2_ levels were measured using the Amplex Red Hydrogen Peroxide Assay Kit (Molecular Probes, Invitrogen, Eugene, OR, USA), following the manufacturer’s protocol. To detect H_2_O_2_ released from kidney tissue and treated HK cells, cell lysate or culture media were reacted with the Amplex Red Reagent in the presence of horseradish peroxidase to produce the red-fluorescent oxidation product resorufin. The fluorescence of resorufin was determined at 530nm excitation and 590 nm emission using a fluorescence microplate reader (Molecular Devices, Sunnyvale, CA, USA). The concentrations of H_2_O_2_ were calculated using standard curves. The loading buffer was measured and subtracted from each value in order to exclude background fluorescence.

### 4.7. Intracellular ROS Measurement

Intracellular ROS generation was measured using 2′,7′-dichlorofluorescein diacetate (DCF-DA). HK-2 cells and kidney tissues were stained for 40 min with 10 µM 2′,7′-dichlorodihydrofluorescein diacetate (H_2_DCFDA; Molecular Probes) in a black 96-well plate. DCF-DA is hydrolyzed by esterases to dichlorofluorescein (DCF), which is trapped within the cell. Then cellular oxidants oxidize this non-fluorescent molecule to fluorescent dichlorofluorescein (DCF). Fluorescence signal intensity was measured at 480 nm excitation and 520 nm emission using a fluorescence microplate reader (Molecular Devices). The value of the fluorescence signal was expressed as a percentage of the control.

### 4.8. Determination of Intracellular Total Cholesterol

From the kidney tissues of ApoE mice, we extracted cellular lipids by chloroform:methanol extraction (4:2:3, chloroform:methanol:water). Total cholesterol levels were determined using a commercially available kit (Cell Biolabs Inc., San Diego, CA, USA) following the manufacturer’s protocol.

### 4.9. Transfection of HK-2 with XDH siRNA

ON-TARGETplus SMARTpool siRNAs used for silencing expression of human XDH genes (ID:7498) and non-targeting (negative control) siRNA were purchased from Dharmacon (Chicago, IL, USA). Four target sequences in human XDH are 5′- AGA GUG AGG UUG ACA AGU U -3′, 5′- GGA GUA ACA UAA CUG GAA U -3′, 5′- UAG AGG AGC UAC ACU AUU C -3′ and 5′- ACA CGG AGA UUG GCA UUG A -3′. siRNAs were used at a concentration of 20 nM. Transfection was performed using Opti-MEM™ transfection medium and Lipofectamine™ (both from Invitrogen, Paisley, UK). One day prior to transfection, HK-2 cells were seeded and cultured to reach 30–40% confluence on the following day. RNAi duplexes for XDH were mixed with Lipofectamine, forming a transfection complex that was added to the plated cells. After 24 h of incubation, the medium was replaced with RPMI, and cells were starved for 6 h. Transfected cells were used for quantitative real-time reverse transcription-polymerase chain reaction (qRT-PCR).

### 4.10. Quantitative Real-Time Polymerase Chain Reactions

Quantitative real-time RT-PCR analysis was performed as described previously [7]. Total RNA was extracted from cell lysates using TRIreagent (Thermo Fisher Scientific, Waltham, MA, USA) according to the provider’s instructions. One microgram of total RNA was reverse transcribed to cDNA using the PrimeScript cDNA synthesis kit (TaKaRa, Otsu, Japan). Quantitative real-time RT-PCR was performed on the ABI PRISM 7700 Sequence Detection System (Applied Biosystems, Foster city, CA, USA) using the SYBR green PCR Master Mix (Applied Biosystems, Foster City, CA, USA). The results were analyzed using the comparative Ct method for relative quantification of gene expression. The primer sets used in this study are listed in Table 1. 

### 4.11. Immunoblot Analysis

Protein concentration was measured using Bradford’s method in lysates of treated HK2 cells and tissues. Total protein (20 µg) was separated by 10% SDS-polyacrylamide gel electrophoresis and transferred to a nitrocellulose membrane. The membrane was blocked with 10% skim milk for 1 h at room temperature, and incubated overnight at 4 °C with primary antibodies. The membrane was incubated with HRP-conjugated polyclonal goat anti-rabbit IgG P0447 or goat anti-mouse IgG p0448 (Dako, Glostrup, Denmark) as the secondary antibody for 1 h and detected using advanced ECL reagents (Amersham Bioscience, Piscataway, NJ, USA). The target protein bands were normalized to that of GAPDH. Expression levels were estimated using Scion Image software (Scion, Frederick, MD, USA). The primary antibodies that detect proteins are listed in Table 2. 

### 4.12. Statistical Analysis

Data are presented as mean ± SEM. Statistical analyses were performed using GraphPad Prism 5.01 (GraphPad Software Inc., La Jolla, CA, USA). The difference among the groups was analyzed using a one-way nonparametric ANOVA followed by Tukey’s multiple comparison test. A *p* value of <0.05 was considered statistically significant.

## Figures and Tables

**Table 1:** Oligonucleotide primer sequences.

Gene	Forward Primer (5′–3′)	Reverse Primer (5′–3′)
**Mouse *Msr1***	CAC GGG ACG CTT CCA GAA T	TGG ACT GAC GAA ATC AAG GAA TT
**Mouse *Scarb1***	GGC CTG TTT GTT GGG ATG AA	CGT TCC ATT TGT CCA CCA GAT
**Mouse *Lcat***	AGC CTT GGC TGT CTG CAT GT	CCCGAGAGAGATAAAACCATCAA
**Mouse *Srebf1***	GGC TAT TCC GTG AAC ATC TCC TA	ATC CAA GGG CAT CTG AGA ACT C
**Mouse ** ***Srebf*** ***2*** ****	GGT CCT CCA TCA ACG ACA AAA T	TAA TCA ATG GCC TTC CTC AGA AC
**Mouse ** ***Nr1h3*** ****	GAG TGT CGA CTT CGC AAA TGC	CCT CTT CTT GCC GCT TCA GT
**Mouse ** ***Abca1*** ****	GGC AAT GAG TGT GCC AGA GTT A	TAG TCA CAT GTG GCA CCG TTT T
**Mouse ** ***Ldlr***	CCAAATGGCATCACACTAGATCTT	CCGATTGCCCCCATTG
**Mouse ** ***Hmgcr*** ****	GGG CCC CAC ATT CAC TCT T	GCC GAA GCA GCA CAT GAT CT
**Mouse ** ***Il-1β*** ****	TCG TGC TGT CGG ACC CAT AT	GGTTCTCCTTGTACAAAGCTCATG
**Mouse ** ***Nlrp3*** ****	TCTCCCGCATCTCCATTTGTA	CGC GCG TTC CTG TCC TT
**Mous ** ***Il-18*** ****	GACAACTTTGGCCGACTTCAC	TCCTCGAACACAGGCTGTCTT
**Mouse ** ***Acta2*** ****	CTGACAGAGGCACCACTG	CATCTCCAGAGTCCAGCA
**Mouse ** ***Cdh***	GCAGTTCTGCCAGAGAAACC	TGGATCCAAGATGGTGATGA
**Mouse ** ***Fn1*** ****	CCA TTC TCC TTC TTC AAG TTT GC	AGG AAT GGC TGT CAG GAT GGT
**Mouse ** ***Col1***	ACA ACC GCT TTG CCA CTT CT	CGT AAG TCA CGG GCA CGT T
**Mouse ** ***Gapdh*** ****	TAA AGG GCA TCC TGG GCT ACA CT	TTA CTC CTT GGA GGC CAT GTA GG
**Human ** ***LDLR***	AGT TGG CTG CGT TAA TGT GAC A	TCT CTA GCC ATG TTG CAG ACT TTG
**Human ** ***SREBF1***	GCT CCT CCA TCA ATG ACA AAA TC	TGC AGA AAG CGA ATG TAG TCG AT
**Human ** ***SREBF2***	AGG CGG ACA ACC CAT AAT ATC A	CTT GTG CAT CTT GGC GTC TGT
**Human ** ***ABCA1***	GAC ATC GTG GCG TTT TTG G	CGA GAT ATG GTC CGG ATT GC
**Human ** ***HMGCR***	GGA CAG GAT GCA GCA CAG AA	GCATGGTGCAGCTGATATATAAATCT
**Human ** ***NR1H3***	CAC CTA CAT GCG TCG CAA GT	CAG GCG GAT CTG TTC TTC TGA
**Human ** ***NOX1***	TGCCTAGAAGGGCTCCAAAC	ACATTCAGCCCTAACCAAACAAC
**Human ** ***NOX2***	AGGGTCAAGAACAGGCTAAGGA	TTCTCCACCTCCAACCCTCTTT
**Human ** ***p47**^**phox**^*	GGCAGGACCTGTCGGAGAA	ATCGCCCCTGCCTCAATAG
**Human ** ***p22**^**phox**^*	ACTTTGGTGCCTACTCCATTGTG	TGTCCCCAGCGCTCCAT
**Human ** ***XDH***	GAAGGCCATCTATGCATCGAA	GAAGGCCATCTATGCATCGAA
**Human ** ***GAPDH***	TTCACCACCATGGAGAAGGCT	TGGTTCACACCCATGACGAAC

**Table 2:** List of antibodies used in immunohistochemistry and immunoblotting.

Antibodies	Cat. No.	Company
**LXRα**	Ab176323	abcam
**HMGCR**	Ab174830	abcam
**ABCA1**	Ab18180	abcam
**α-SMA**	Ab5694	abcam
**Fibronectin**	Ab2413	abcam
**IL-1β**	12242	Cell signaling
**NLRP3**	13158	Cell signaling
**NF-kappaB p65**	8242S	Cell signaling
**NF-kappaBphos-p65**	3036S	Cell signaling
**NF-kappaBp105/p50**	3035S	Cell signaling
**GAPDH**	2118S	Cell signaling

## Abbreviations

ABCA1	ATP Binding Cassette Subfamily A Member 1
ABCG1	ATP-binding cassette super-family G Member 1
ApoE KO	Apolipoprotein E Knockout
BUN	Blood urea nitrogen
CD68	Cluster of Differentiation 68
CKD	Chronic kidney disease
DCFDA	Dichlorodihydrofluorescein diacetate
HC	High-cholesterol
HMGCR	3-Hydroxy-3-Methylglutaryl-CoA Reductase
Il-1	Interleukin-1
LCAT	Lecithin-cholesterol acyltransferase
LDL	Low-density lipoprotein
LDLR	Low density lipoprotein receptor
LXRα	Liver X receptor α
MSR1	Macrophage scavenger receptor types I
NR1H3	Nuclear receptor subfamily 1 group H member 3
NADPH	Nicotinamide adenine dinucleotide phosphate
NF-κB	Nuclear Factor-kappaB
NLRP3	NOD-, LRR- and pyrin domain-containing protein 3
NOX	NADPH oxidase
PAS	Periodic acid-Schiff
ROS	Reactive oxygen species
siXDH	Small interfering RNA against XDH
SCARB1	Scavenger receptor class B type 1
SREBF	Sterol regulatory element binding transcription factor
TC	Total cholesterol
TG	Triglyceride
TNF-α	Tumor necrosis factor α
TP	Topiroxostat
XO	Xanthine oxidase
α-SMA	Alpha-smooth muscle actin
